# 105. Burden and Epidemiology of Human Metapneumovirus (hMPV) Acute Respiratory Infection (ARI) among American Indian and Alaska Native (AI/AN) Children <5 years of Age, November 2019-May 2024

**DOI:** 10.1093/ofid/ofaf695.040

**Published:** 2026-01-11

**Authors:** Rachel Hartman, Sainimere Boladuadua, Catherine Sutcliffe, James Keck, Amanda Burrage, Angela Campbell, James Chappell, Fatimah S Dawood, Christine Desnoyers, Jennifer Dobson, Joel Espinoza, Natasha B Halasa, Verlena Little, James B McAuley, Meredith L McMorrow, Kelly Menachof, Linda Oxley, Dennie Parker Riley, Mila M Prill, Kim Taylor, Rosalyn Singleton, Laura Hammitt

**Affiliations:** Johns Hopkins Bloomberg School of Public Health, Baltimore, Maryland; Johns Hopkins Bloomberg School of Public Health, Baltimore, Maryland; Johns Hopkins, Baltimore, MD; Alaska Native Tribal Health Consortium, Anchorage, Alaska; Tuba City Regional Health Care Corporation, Tuba City, Arizona; Emory University School of Medicine / CDC; Vanderbilt University Medical Center, Nashville, Tennessee; CDC, Atlanta, Georgia; Yukon-Kuskokwim Health Corporation, Yukon Kuskokwim Delta, Alaska; Alaska Native Tribal Health Consortium, Anchorage, Alaska; Johns Hopkins Bloomberg School of Public Health, Baltimore, Maryland; Vanderbilt University Medical Center, Nashville, Tennessee; Johns Hopkins Bloomberg School of Public Health, Baltimore, Maryland; Indian Health Service, Whiteriver, AZ; CDC/NCIRD/CORVD/SPB, Atlanta, GA; Chinle Service Unit, Navajo Area Indian Health Service, Chinle, Arizona; Alaska Native Tribal Health Consortium, Anchorage, Alaska; Johns Hopkins Bloomberg School of Public Health, Baltimore, Maryland; Centers for Disease Control & Prevention, Atlanta, GA; Johns Hopkins Bloomberg School of Public Health, Baltimore, Maryland; Alaska Native Tribal Health Consortium, Anchorage, Alaska; Johns Hopkins School of Public Health, Baltimore, Maryland

## Abstract

**Background:**

hMPV is a common cause of medically attended ARI (MA-ARI) in US children aged < 5 years, but few data are available on disease burden in AI/AN children. For respiratory syncytial virus (RSV), another common cause of pediatric respiratory illness, hospitalization rates in AI/AN children < 5 years historically have been higher than for the general US population < 5 years (17-40/1000 in some remote AI/AN communities versus 4-6/1000). We describe the epidemiology of medically attended hMPV-associated ARI in AI/AN children and estimate incidence rates of hMPV-associated hospitalizations.

**Figure:**
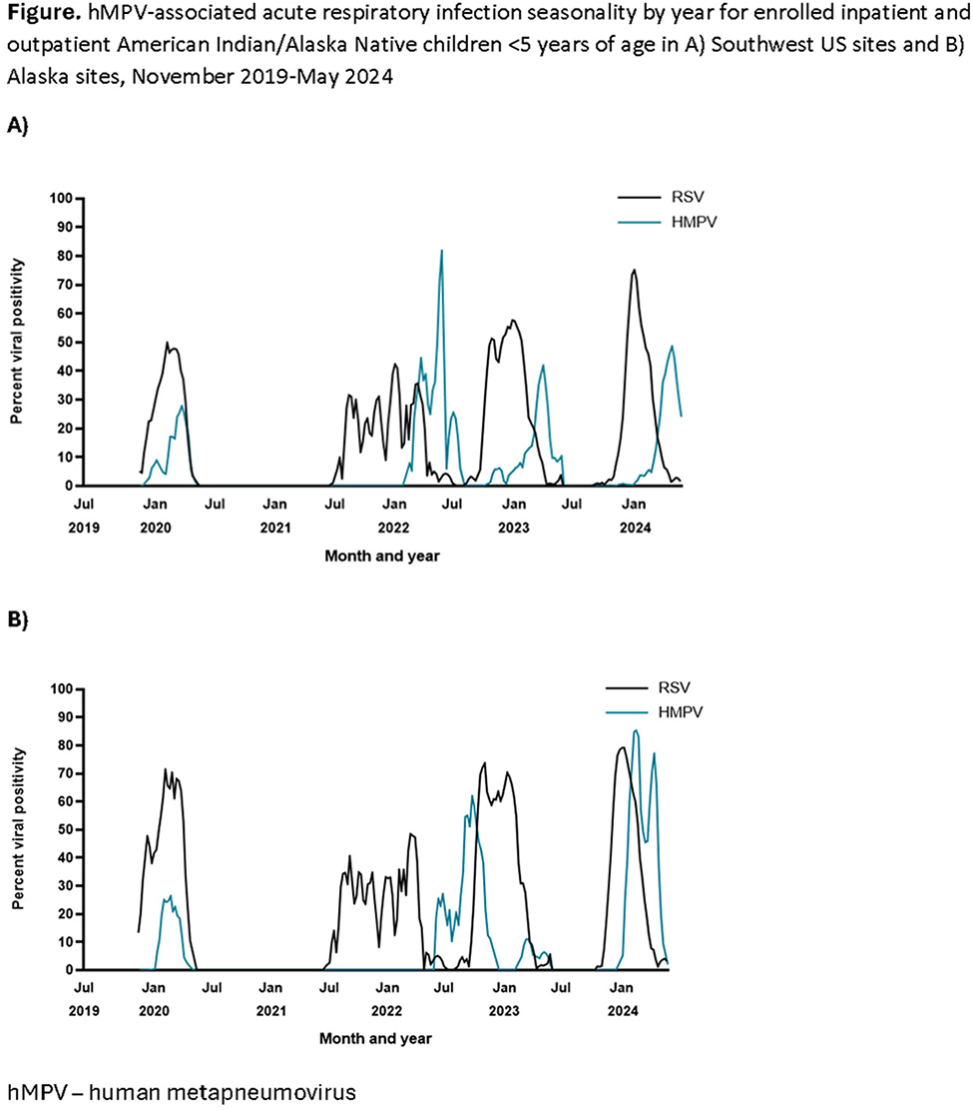

**Figure:**
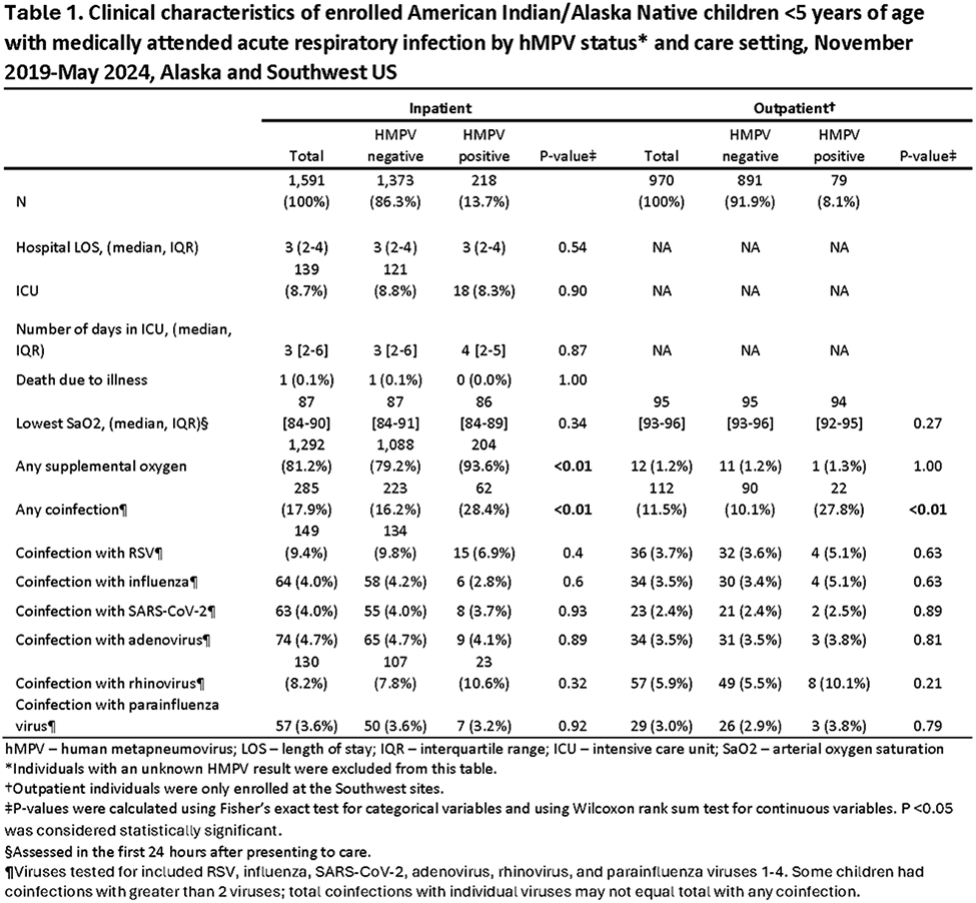

**Methods:**

We conducted population-based surveillance for MA-ARI in hospitalized and outpatient AI/AN children aged < 5 years in the Southwest US (Navajo Nation and White Mountain Apache Tribal lands) and Alaska (Yukon Kuskokwim Delta and Anchorage) during November 2019-May 2024. Nasal swabs were tested by PCR for hMPV and RSV. We described the seasonality of hMPV and RSV detection and compared the clinical presentation of children with hMPV-positive versus hMPV-negative MA-ARI using Fisher’s exact test and Wilcoxon rank sum test. We used Poisson regression to estimate annual hMPV-associated hospitalization incidence per 1000 children stratified by age and site.

**Figure:**
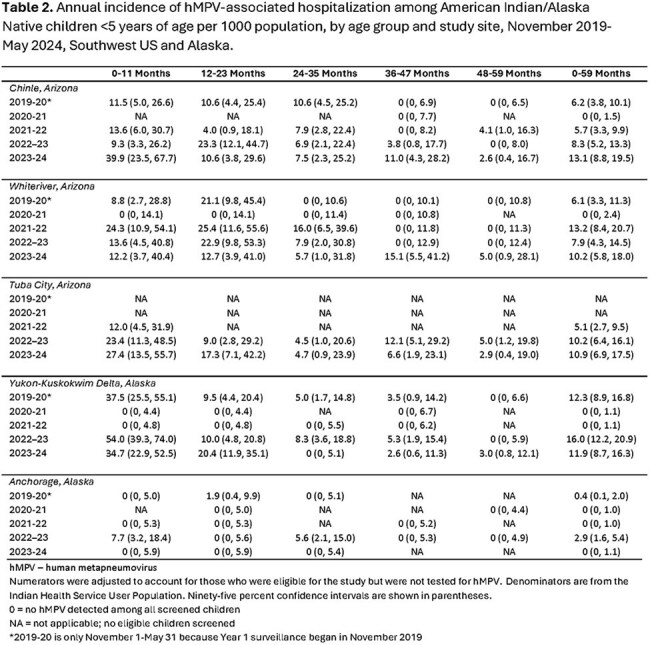

**Results:**

hMPV seasonality varied regionally but circulation typically followed the onset of the RSV season; the COVID-19 pandemic disrupted circulation during 2021 (Fig). Among the 2561 (90%) of 2836 enrollees with hMPV testing, hMPV was detected in 14% (1373/1591) of swabs from inpatient children and 8% (79/970) of swabs from outpatient children (Table 1). Clinical characteristics were generally similar in children with hMPV-positive compared to hMPV-negative MA-ARI. The incidence of HMPV-associated ARI hospitalization among children < 5 years ranged from 0-16.0/1000 and age-group patterns varied by site and year (Table 2).

**Conclusion:**

hMPV-associated hospitalization incidence rates in AI/AN children < 5 years were lower than historical RSV-associated hospitalization rates in these same communities but similar to or greater than historical RSV rates in the general US child population. These findings provide a baseline for hMPV disease burden for assessments of future prevention products.

**Disclosures:**

All Authors: No reported disclosures

